# Artificial intelligence for traumatic brain injury imaging: a translational review from algorithm development to clinical implementation

**DOI:** 10.3389/fneur.2026.1798780

**Published:** 2026-03-17

**Authors:** Yansong Xu, Cuiqing Huang

**Affiliations:** Department of Emergency, The First Affiliated Hospital of Guangxi Medical University, Nanning, Guangxi, China

**Keywords:** artificial intelligence, clinical translation, neuroimaging analysis, precision medicine, traumatic brain injury

## Abstract

**Background:**

Traumatic brain injury (TBI) remains a major global health burden, with computed tomography (CT) serving as the frontline imaging modality for acute assessment. However, CT interpretation is hampered by subjectivity, oversight in busy emergency settings, and limited prognostic accuracy of traditional scoring systems. Artificial intelligence (AI), particularly deep learning, offers transformative potential to automate and enhance TBI neuroimaging analysis.

**Main body:**

This review systematically synthesizes the translational pathway of AI in TBI imaging, from algorithm development to clinical implementation. AI models, especially convolutional neural networks, demonstrate high performance (sensitivity up to 96%) in detecting and classifying intracranial hemorrhage, segmenting lesions, and automating radiological scoring. Through multimodal data fusion, AI further shows promise in predicting patient outcomes, from near-term mortality to long-term functional recovery. Beyond pattern recognition, AI-derived imaging biomarkers hold potential as surrogate endpoints in therapeutic trials. However, prospective and real-world validation studies reveal a critical evidence gap: while AI tools improve diagnostic metrics and workflow efficiency, robust randomized controlled trials demonstrating direct improvement in patient-centered outcomes are still lacking.

**Conclusion:**

AI is poised to revolutionize TBI neuroimaging by increasing diagnostic objectivity, efficiency, and prognostic precision. Successful clinical translation, however, requires overcoming key challenges related to data heterogeneity, model interpretability, and workflow integration. Future efforts must prioritize the generation of high-quality multi-center datasets, the development of explainable AI, and—most critically—the execution of prospective trials with patient outcome endpoints. Collaborative, interdisciplinary research is essential to translate these technological advances into tangible improvements in TBI care and recovery.

## 1 Introduction

Traumatic brain injury (TBI) poses a severe global public health challenge, with over 60 million people experiencing TBI annually, and its incidence is on the rise (1). Approximately 25% of injury-related deaths are associated with TBI (2). Even survivors may face a spectrum of consequences, ranging from short-term non-disabling impairments to long-term severe sequelae (3). In the acute phase management of TBI, computed tomography (CT) plays an indispensable role. Due to its rapid scanning speed, high accessibility, and sensitivity to acute hemorrhage, CT has become the preferred initial imaging modality and serves as the cornerstone for rapid decision-making, such as determining the need for neurosurgical intervention (4, 5). Despite its critical importance, the clinical interpretation of CT scans faces multiple challenges. Firstly, interpretive subjectivity is widespread, as significant discrepancies in the assessment of intracranial injuries can exist among radiologists with different levels of seniority and experience (4). Secondly, information overload in emergency department settings may lead to the oversight of subtle yet clinically significant lesions (6). Finally, traditional prognostic prediction models (e.g., the IMPACT score), which are based on imaging and clinical indicators such as the Glasgow Coma Scale (GCS), have inherent limitations (7).

**Figure 1:**
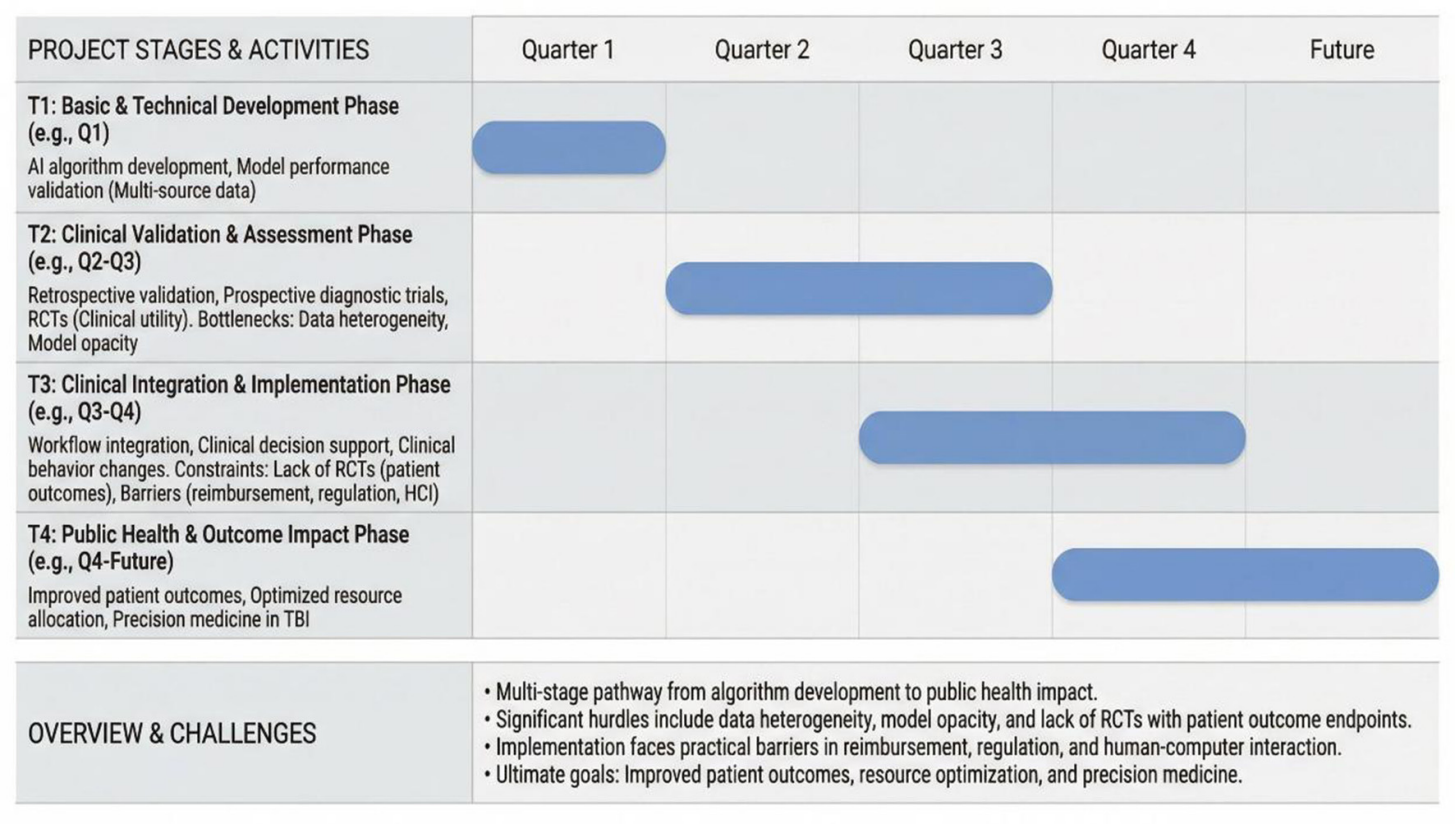
Translational pathway for artificial intelligence in traumatic brain injury neuroimaging.

Artificial intelligence (AI), particularly deep learning technologies, presents new opportunities to address these challenges. AI models enable automated, rapid, and objective quantitative analysis of CT and MRI images. They excel at identifying complex imaging patterns that are difficult for the human eye to discern, providing a powerful tool for discovering novel imaging biomarkers and enhancing the accuracy of prognostic prediction (8). This review aims to systematically elaborate on the latest advancements of artificial intelligence in the field of neuroimaging for traumatic brain injury. Starting from core technical models and data foundations, it delves into its applications in injury identification, quantitative analysis, and prognostic prediction, exploring its extensive clinical applications. Finally, it discusses current challenges and future directions (Figure 1).

This article is a narrative review, not a systematic review or meta-analysis. It aims to provide a conceptual and translational overview of the application of artificial intelligence in traumatic brain injury neuroimaging, rather than a comprehensive synthesis of all available evidence. To ensure transparency, we briefly outline our literature identification process. The peer-reviewed articles covered in this review were primarily identified through targeted searches in the PubMed and Web of science databases. The search focused on English-language literature published between January 2015 and December 2025, a period witnessing the rapid evolution of deep learning in medical imaging. Key search terms included combinations of “artificial intelligence,” “deep learning,” “machine learning,” “traumatic brain injury,” “neuroimaging,” “computed tomography,” “intracranial hemorrhage,” and “prognosis.” The selection of references was guided by thematic relevance to the translational pathway (from algorithm development to clinical implementation), methodological quality (prioritizing large-cohort studies, systematic reviews, and meta-analyses where available), and their contribution to illustrating key concepts, challenges, and future directions within the narrative.

## 2 AI models and data foundation

In the field of neuroimaging, the application of AI models has significantly improved the efficiency and accuracy of diagnosing critical conditions such as intracranial hemorrhage (9). The core technologies primarily encompass traditional machine learning and deep learning. Traditional machine learning methods, such as Support Vector Machines (SVM) and Random Forests, rely on expert-defined manual extraction of imaging features (e.g., texture, shape) for model training. For instance, Turcato et al. (10) utilized a decision tree algorithm to predict the risk of intracranial hemorrhage in patients with mild traumatic brain injury taking anticoagulants, demonstrating good clinical applicability. However, the limitations of manual feature extraction have propelled deep learning to the forefront. The Convolutional Neural Network (CNN), as the core architecture, excels in tasks such as image classification (e.g., determining the presence or absence of hemorrhage), object detection (e.g., locating hematomas), and segmentation (e.g., precisely delineating lesion contours). A systematic review and meta-analysis by Daugaard et al. (11) showed that CNNs can achieve a pooled sensitivity of up to 92% in detecting intracranial hemorrhage, performing comparably to radiologists. For sequential imaging or when combined with clinical time-series data, Recurrent Neural Networks (RNN) and Transformer models can capture dynamic changes, such as analyzing the evolution of blood clots in stroke patients during follow-up (12).

High-quality data serves as the cornerstone for the generalization ability of AI models. Public datasets such as CQ500 and the RSNA Intracranial Hemorrhage Detection Challenge dataset provide critical resources for algorithm development. A systematic review by Agarwal et al. (13) pointed out that while these datasets facilitate model comparison and validation, they suffer from limitations such as homogeneous sample sources and label noise, which may impact their generalization performance on external cohorts. Data standardization is another crucial step. The heterogeneity in images caused by different CT scanners and acquisition protocols (e.g., variations in resolution and contrast) poses challenges to model stability. Preprocessing steps such as normalization and resampling can partially mitigate inter-scanner variability, thereby enhancing model robustness (14). Furthermore, the quality of data annotation directly influences model performance. Research by Villringer et al. (15) demonstrated that integrating AI algorithms into clinical workflows can significantly improve annotation consistency and diagnostic quality (e.g., increasing hemorrhage detection accuracy to 98%). Commonly used annotation tools, such as ITK-SNAP and 3D Slicer, support precise lesion delineation, providing a reliable foundation for model training (16).

## 3 Application of AI in TBI image recognition and quantitative analysis

CT assessment of traumatic brain injury is a pivotal domain where AI technology demonstrates significant potential. Through automated identification, segmentation, and quantification, AI is profoundly transforming the TBI diagnosis and treatment workflow, aiming to enhance diagnostic efficiency, objectivity, and the accuracy of prognosis prediction (Table 1).

**Table 1:** Summary of AI applications in TBI image recognition and quantitative analysis.

**Detection/analysis target**	**AI method/model type**	**Key performance metrics**	**Representative study/references**
Intracranial hemorrhage detection	Convolutional neural network (CNN)	Pooled sensitivity: 92%−96%; specificity: 95%	Daugaard et al. (11); Karamian et al. (19)
Hemorrhage classification	Deep learning (CNN-based)	High accuracy in subtype identification	Teneggi et al. (17)
Skull fracture detection	Computer-aided detection (CAD)/CNN	Improved detection, reduced missed diagnoses	Liew et al. (20)
Midline shift quantification	Automated segmentation and measurement	Objective measurement, reduces inter-observer variability	Hsu et al. (22)
Hematoma segmentation	3D deep neural network	Dice coefficient: 0.93	Sharrock et al. (16)
Edema segmentation	Semantic segmentation CNN	Accurate delineation of edema regions	Kok et al. (23)
Automated radiological scoring	Machine learning/CNN	Auto-calculation of Marshall, Rotterdam scores	Kazimierska et al. (24)

### 3.1 Detection and classification of injuries

Models based on convolutional neural networks can now efficiently screen head CT scans for abnormal signs. In the detection of intracranial hemorrhage, AI models can not only determine the presence or absence of hemorrhage but also further identify its specific type (17, 18). A systematic review by Karamian et al. (19) indicated that deep learning achieves pooled sensitivity and specificity as high as 96 and 95%, respectively, for detecting intracranial hemorrhage on non-contrast CT scans. Furthermore, AI demonstrates excellent performance in detecting skull fractures. A review by Liew et al. (20) showed that computer-aided techniques can effectively identify linear or depressed fractures, reducing missed diagnoses caused by complex imaging or reader fatigue. Beyond direct injuries, the measurement of midline shift is a crucial indicator for assessing mass effect and guiding clinical decision-making. AI algorithms can achieve rapid and objective quantification of this parameter by automatically localizing the falx cerebri and measuring its displacement distance (21). Hsu et al. (22) noted that this automated quantitative analysis helps eliminate inter-observer variability, providing a reliable tool for rapid triage in the emergency department.

### 3.2 Fine-grained segmentation and quantification of injuries

Pixel-level fine-grained segmentation represents an advanced capability of AI toward achieving precision medicine. This technique enables the voxel-by-voxel delineation of hemorrhage regions, cerebral contusions, and surrounding edema (23). Sharrock et al. (16) developed and validated a 3D deep neural network capable of accurately segmenting intracerebral hematomas, achieving a Dice similarity coefficient of 0.93, thereby providing a reliable tool for monitoring hematoma volume. The direct clinical value of this automated segmentation lies in enabling the precise calculation of hematoma volume, which is a key factor in determining the timing of surgical intervention. Similarly, quantifying the extent of edema aids in assessing the risk of elevated intracranial pressure. A study by Villringer et al. established a hybrid clinical-radiomic model based on fully automated segmentation, which successfully predicted early hematoma expansion in spontaneous intracerebral hemorrhage, demonstrating the potential of quantitative analysis in prognosis assessment (15).

### 3.3 Automated scoring of injury severity

Translating complex imaging findings into standardized severity indicators represents another significant contribution of AI in assisting clinical communication and risk assessment. Currently, studies have successfully trained AI models capable of automatically calculating CT-based radiological scores such as the Marshall and Rotterdam scores (24). These scoring systems synthesize various features, including the compression status of the basal cisterns, the degree of midline shift, and the types of injuries present. A systematic review by Lampros et al. (25) confirmed the effectiveness of machine learning applications in pediatric TBI, among which automated scoring is a core function. Another meta-analysis by Wang et al. (26) further indicated that machine learning models demonstrate excellent performance (AUC = 0.94) in predicting mortality risk in TBI patients, and these models often incorporate the aforementioned automatically calculated radiological scores as important predictive features. This automated scoring not only relieves radiologists from tedious assessments but, more importantly, ensures scoring consistency, providing multidisciplinary teams with objective and reproducible severity benchmarks.

## 4 Integrated application of AI in TBI prognostic prediction

The clinical management of traumatic brain injury requires not only the accurate identification of acute injuries but, more crucially, the precise prediction of individualized patient outcomes. By integrating multi-dimensional data, artificial intelligence is progressively moving beyond mere image recognition to evolve into a powerful prognostic tool, providing a forward-looking basis for clinical decision-making (Table 2).

**Table 2:** Overview of AI-based multimodal prognostic prediction studies in TBI.

**Prediction target**	**Data types integrated**	**AI/modeling approach**	**Performance (AUC/accuracy)**	**Study/references**
In-hospital mortality	CT imaging + clinical variables	Deep learning/fusion model	AUC up to 0.94	Wang et al. (26)
Need for neurosurgery (within 24 h)	CT features + clinical parameters	Machine learning	High predictive accuracy	Moyer et al. (28)
1-year functional outcome	Imaging + demographic + clinical follow-up	Multimodal machine learning	Significant predictive value	Van Deynse et al. (29)
Early hematoma expansion	CT radiomics + clinical data	Hybrid clinical-radiomic model	Effective prediction	Wang et al. (36)
Cognitive outcome prediction	DTI-based tractography + clinical scores	AI-based tract quantification	Correlation with cognitive outcomes	Yeh et al. (30)
Diffuse axonal injury prognosis	MRI features + clinical indicators	Computational pattern analysis	Independent prognostic value	Lin and Yuh (31)

### 4.1 Beyond identification: from imaging to prognosis

TBI patient outcomes are highly heterogeneous, influenced by multiple factors such as the nature of the injury, the patient's baseline status, and treatment response. Therefore, predicting long-term functional outcomes—such as mortality and the degree of neurological recovery—is critical for developing individualized treatment plans, communicating expectations with families, and optimizing healthcare resource allocation (25, 26). Achieving the leap from “what is seen” to “what will happen” represents the inevitable trend and core value of AI application in the TBI field.

### 4.2 Multimodal data fusion strategies

Recent research focuses on multimodal data fusion strategies, constructing model architectures capable of simultaneously processing imaging features and clinical variables. Fusion models demonstrate exceptional performance in predicting mortality risk in TBI patients, achieving a pooled area under the receiver operating characteristic curve (AUC) of up to 0.94, significantly outperforming models relying on a single data source (26). Regarding prediction endpoints, AI models can now effectively predict short-term outcomes, such as in-hospital mortality and the need for neurosurgical intervention (27, 28). More importantly, AI also shows potential in predicting long-term functional outcomes. Research by Van Deynse et al. (29) through analysis of the large-scale CENTER-TBI database, successfully constructed a model predicting outcomes 1 year post-injury, highlighting its long-term value.

### 4.3 Mining imaging biomarkers

Deep learning and radiomics techniques can extract subtle imaging features from medical images that are difficult for the human eye to define. These features may be closely related to underlying pathophysiological processes (such as axonal injury and microcirculation dysfunction), thereby serving as powerful new imaging biomarkers. A review by Yeh et al. (30) on the use of diffusion tensor imaging-based tractography in TBI indicated that AI can quantify the extent of white matter tract damage, which is significantly correlated with patients' cognitive functional outcomes. Lin and Yuh (31) also emphasized that computational analytical methods can identify unique imaging patterns associated with diffuse axonal injury and cerebral edema, which have independent predictive value for prognosis. Through machine learning models, these vast, subtle features are screened and integrated to form predictive signatures strongly correlated with prognostic endpoints, thereby providing objective, quantitative insights that surpass traditional interpretive experience.

Beyond prediction, these AI-derived quantitative imaging biomarkers hold significant translational potential. They could serve as objective, early surrogate endpoints in clinical trials for novel neuroprotective therapies, potentially reducing trial duration and cost. Furthermore, their integration with emerging fluid biomarkers (e.g., GFAP, UCH-L1) represents a powerful multimodal approach to achieving a more comprehensive and biologically grounded assessment of injury severity and recovery potential, epitomizing the “Bedside to Bench and Back” cycle central to translational medicine.

### 4.4 Evidence from clinical trials and real-world validation

The ultimate value of any medical AI tool lies not in its laboratory performance, but in its real-world impact on patient care. While retrospective studies demonstrate that AI models can achieve expert-level diagnostic accuracy, prospective validations reveal a critical translational disconnect: high technical performance does not automatically translate into improved patient outcomes. Understanding why this gap exists is essential.

First, diagnostic metrics are surrogate endpoints. An AI tool with 99% sensitivity for hemorrhage detection may not change patient outcomes if the finding was clinically obvious, if it does not alter management, or if it leads to unnecessary interventions. As shown in Table 3, excellent algorithm performance does not equate to faster treatment or better recovery (11, 19). Second, workflow integration is a socio-technical challenge. AI output must be seamlessly integrated into radiology workflows and PACS at the right time and to the right decision-maker. An alert that appears after a report is finalized, or that contributes to “alert fatigue,” provides no clinical value. Third, AI informs but does not dictate clinical decisions. The threshold for neurosurgical intervention depends on patient factors, surgeon judgment, and risk tolerance. As Fussell et al. (8) demonstrated, AI recommendations are interpreted differently by trainees vs. experts. A perfect prediction may not alter behavior if it conflicts with clinical heuristics. Finally, health system constraints are the ultimate gatekeepers. An AI tool that accelerates diagnosis is irrelevant if no neurosurgeon or operating room is available. The true impact of AI is contingent on the system's capacity to translate insights into action.

**Table 3:** Evidence levels and translational gaps for AI in TBI neuroimaging: from diagnostic accuracy to patient outcomes.

**Type of evidence/study design**	**Representative study**	**Primary aspect evaluated**	**Key findings/strength of evidence**	**Current translational gaps/limitations**
Retrospective diagnostic accuracy study	Daugaard et al. (11) Karamian et al. (19)	**Algorithm performance**: sensitivity, specificity, dice coefficient, etc.	Demonstrates that AI models can achieve or surpass radiologist-level performance in distinguishing cases from controls (sensitivity >92%).	**Lacks clinical context**: evaluated on curated, controlled datasets without integration into real clinical workflows. Cannot demonstrate impact on clinical decisions or outcomes.
Prospective diagnostic validation study	Villringer et al. (15)	**Diagnostic quality in clinical setting**: detection accuracy and consistency after integrating AI into real-world emergency workflow.	AI assistance significantly improved the quality and consistency of acute intracranial hemorrhage detection on CT (accuracy increased to 98%).	**Limitation of surrogate endpoints**: primary endpoints remain diagnostic accuracy, not time-to-treatment or patient prognosis. Demonstrates feasibility of workflow integration but not improvement in final outcomes.
Randomized controlled trial (RCT)/prospective utility study	Fussell et al. (8)	**Human-AI interaction and clinical utility**: improvement in diagnostic efficacy for users with different experience levels.	AI provided greater benefit to junior trainees than to expert radiologists, revealing that clinical utility is context- and user-dependent.	**Limited patient-centered endpoints**: study focus remains on diagnostic performance (sensitivity), not patient-centered outcomes (e.g., mortality, functional recovery).
Predictive model and decision support study	Moyer et al. (28)	**Impact on clinical decision-making**: predicting the need for neurosurgical intervention to optimize triage and resource preparation.	The model effectively predicted the risk of requiring neurosurgery within 24 h in moderate-to-severe TBI patients, showing potential for decision support.	**Lack of prospective intervention trials**: model performance is largely validated on retrospective data. No RCT has proven that using the model reduces time-to-surgery or improves outcomes in surgical patients.
RCT demonstrating improved patient outcomes (current gap)	**None reported for TBI-AI imaging tools to date**	**Patient-centered outcomes**: mortality, neurological functional scores (e.g., GOS-E), hospital length of stay, quality of life.	**Evidence gap**: currently, no rigorously designed RCT has demonstrated that using an AI imaging tool directly leads to statistically significant improvement in TBI patient outcomes.	**The major translational chasm**: this is the essential evidence threshold for an AI tool to transition from an “aid” to a “component of standard care.” Future studies must be designed with such hard endpoints as primary goals.

## 5 Challenges and future directions

Although artificial intelligence demonstrates significant potential in the imaging analysis of traumatic brain injury, its transition from research to widespread clinical practice still faces multiple challenges.

### 5.1 Data-level challenges

High-quality data is the cornerstone of AI model training, yet significant bottlenecks persist. Firstly, issues such as small dataset sizes and inconsistent annotation quality are common. Secondly, the challenge of data heterogeneity is severe. Different medical institutions use various CT/MRI scanners, imaging protocols, and parameters, leading to differences in imaging feature distributions (8). Additionally, patient privacy and ethical concerns involved in multi-center data sharing pose significant obstacles to data aggregation.

### 5.2 Model-level challenges

At the model level, the primary issue is the “black box” nature and interpretability. The decision-making process of deep learning models is often opaque, while clinicians need to establish trust in AI's judgments to adopt its recommendations. To address this, Explainable AI (XAI) methods, such as generating attention maps or Grad-CAM, are widely used to visualize the image regions upon which the model bases its decisions (32). Another key challenge is generalizability and robustness. Models developed in ideal environments often lack guaranteed stability when faced with real-world images from diverse sources and of varying quality. Zaidi et al., in an AI competition evaluation, pointed out that model performance fluctuates significantly across different external validation sets, highlighting the necessity for enhanced robustness (33).

### 5.3 Clinical translation and implementation challenges

Successfully integrating AI models into the clinical workflow is a crucial step toward realizing their value, but the process is complex. First, regarding regulatory approval, the FDA 510 (k) pathway may inadequately assess the risks of continuously learning AI systems (33), while the EU MDR and the forthcoming AI Act impose more stringent requirements for clinical evidence. Second, cost-effectiveness and reimbursement mechanisms are critical bottlenecks: AI implementation involves software, hardware, and maintenance costs, yet specific billing codes are lacking, and health economic evaluations remain scarce (13). Third, domain shift poses a significant challenge—model performance may degrade substantially when applied to new equipment or populations, a concern particularly acute in TBI where imaging protocols are not fully standardized across centers (33). Finally, human-AI interaction studies demonstrate that AI utility varies with user experience, and building clinical trust requires explainability and consistently reliable performance (8). Systematically addressing these barriers is a prerequisite for successful clinical translation of AI. In summary, the path to clinical implementation is not a simple linear progression from validation to adoption. It requires navigating a complex ecosystem of regulatory requirements, economic constraints, technical robustness, and human factors. Recognizing and proactively addressing these barriers is essential for translating the promise of AI in TBI neuroimaging into tangible improvements in patient care.

### 5.4 Future directions

To address the aforementioned challenges, future efforts should focus on the following directions: First, collaboratively developing larger, high-quality, annotated datasets with multi-center representation to train more robust models. Second, continuously advancing the development of interpretable and trustworthy AI models to enhance clinicians' understanding and trust. Third, actively conducting prospective randomized controlled trials, similar to the “direct-to-angiography suite” trial conducted by Sarraj et al. (34) to provide evidence of the practical impact of AI tools on clinical decision-making speed and patient outcomes. Finally, moving beyond single imaging modalities to explore deeper multimodal fusion—integrating CT, MRI sequences with clinical data, and even blood biomarkers—to build more powerful diagnostic and predictive models. This will be key to achieving truly individualized precision medicine (35).

### 5.5 Methodological limitations in current AI research

Beyond the translational barriers discussed above, the AI literature in TBI neuroimaging is itself constrained by fundamental methodological limitations that warrant critical appraisal. First, overfitting remains a pervasive concern. Many models are optimized to perform exceptionally well on their training data but fail to generalize because they have learned noise rather than true signal—a risk amplified when sample sizes are small relative to model complexity (11). Second, limited external validation severely undermines claims of generalizability. Most studies validate models using internal splits from a single institution, which cannot capture the full spectrum of scanner variability, protocol differences, and population diversity encountered in real-world practice (13). When external validation is performed, performance often drops substantially, revealing fragile models. Third, dataset homogeneity introduces systematic bias. Publicly available datasets such as CQ500 and RSNA, while invaluable for algorithm development, predominantly originate from specialized centers and may underrepresent certain demographic groups, injury types, or imaging protocols (13). Models trained on these data risk performing inequitably when deployed in underserved or diverse populations. Finally, publication bias distorts the evidentiary landscape. Studies reporting positive results—high accuracy, impressive AUCs—are more likely to be published than those reporting negative or null findings, creating an inflated perception of AI readiness (19).

## 6 Conclusion

Artificial intelligence stands at the forefront of a revolution in TBI neuroimaging, offering capabilities that span the entire clinical continuum from rapid, automated injury detection to precise volumetric quantification and data-driven prognostic prediction. These tools hold the promise of serving as powerful adjuncts to clinical expertise, enhancing both the efficiency and objectivity of patient assessment. However, their successful translation into routine practice hinges on far more than algorithmic refinement. It demands sustained, synergistic collaboration across disciplines—uniting data scientists, radiologists, neurosurgeons, intensivists, and emergency physicians. By collectively addressing the core translational challenges of data quality, model interpretability, clinical validation, and workflow integration, the community can steer these promising technologies toward their ultimate goal: tangibly improving the care pathways and long-term outcomes for patients suffering from traumatic brain injury.
